# The Role of the Glucocorticoids in Developing Resilience to Stress and Addiction

**DOI:** 10.3389/fpsyt.2013.00068

**Published:** 2013-08-01

**Authors:** Subhashini Srinivasan, Masroor Shariff, Selena E. Bartlett

**Affiliations:** ^1^Ernest Gallo Clinic and Research Center at the University of California San Francisco, Emeryville, CA, USA; ^2^Translational Research Institute and Institute for Health and Biomedical Innovation, Queensland University of Technology, Brisbane, QLD, Australia

**Keywords:** addiction, glucocorticoid, stress, resilience, cholinergic, nicotinic acetylcholine receptors, mifepristone, orexin

## Abstract

There is emerging evidence that individuals have the capacity to learn to be resilient by developing protective mechanisms that prevent them from the maladaptive effects of stress that can contribute to addiction. The emerging field of the neuroscience of resilience is beginning to uncover the circuits and molecules that protect against stress-related neuropsychiatric diseases, such as addiction. Glucocorticoids (GCs) are important regulators of basal and stress-related homeostasis in all higher organisms and influence a wide array of genes in almost every organ and tissue. GCs, therefore, are ideally situated to either promote or prevent adaptation to stress. In this review, we will focus on the role of GCs in the hypothalamic-pituitary adrenocortical axis and extra-hypothalamic regions in regulating basal and chronic stress responses. GCs interact with a large number of neurotransmitter and neuropeptide systems that are associated with the development of addiction. Additionally, the review will focus on the orexinergic and cholinergic pathways and highlight their role in stress and addiction. GCs play a key role in promoting the development of resilience or susceptibility and represent important pharmacotherapeutic targets that can reduce the impact of a maladapted stress system for the treatment of stress-induced addiction.

## Introduction

Susceptibility to developing an addiction is governed by genetics and modified by experience and the environment. Stress plays an important role in increasing susceptibility to addiction. McEwen eloquently wrote that, “human lifetime experiences have a profound impact on the brain, both as a target of stress and allostatic load/overload and as a determinant of physiological and behavioral response to stressors” (1). The ability to cope with stress or resilience (the capacity to bounce back following adversity) significantly predicts whether a person will subsequently develop a stress-related neuropsychiatric disease such as anxiety, depression, and addiction [reviewed in (2)]. A large majority of population have experienced a traumatic event during their lifetime. However, only a small percentage will subsequently experience chronic distress leading to post-traumatic stress disorder (PTSD) or addiction to alcohol or other drugs (3). In most cases, however, people have resilience and do not develop a disease or disorder following exposure to stressors. The emerging field of the neuroscience of resilience is uncovering new circuits and molecules that serve to protect against stress-related neuropsychiatric diseases.

It has often been assumed that resilience is an innate or passive mechanism that cannot be changed. However, research in animals and humans suggest that developing resilience may be a learnt behavior (2). Individuals have the capacity to learn to be resilient by developing mechanisms that protect from the maladaptive effects of stress. Glucocorticoids (GCs), cortisol in humans, or corticosterone in rodents are important regulators of basal and stress-related homeostasis and have been shown to modulate an array of genes in many organs and tissues (4–6). Thus, GCs are ideally placed to regulate a multitude of signaling pathways activated in response to stress and addiction. In this review, we will focus on the role of GCs in the hypothalamic-pituitary adrenocortical (HPA) axis in regulating basal and chronic stress responses. In addition, we will focus on two systems, the orexinergic and cholinergic systems and their roles in mediating stress and addiction. We will further discuss the emerging interaction between these systems with GCs and in regulation of stress. Lastly, as GCs play a key role in promoting either resilience or susceptibility to stress, we will examine the pharmacotherapeutic opportunities that target GCs for the treatment of stress-induced addiction.

## The Role of the HPA Axis and the Glucocorticoids in the Neurobiology of Resilience to Stress

The mechanisms that govern an organism’s ability to handle stress has been well described in microorganisms that have specialized hubs, called stressosomes, that govern responses to an array of physical and environmental insults (7, 8). The stressosome is a unique structure within the microorganism that precisely orchestrates the molecular machinery that tunes the magnitude of the response to a stressor. The stressosome ultimately ensures the survival of the cell in response to an extensive variety of chemical and physical stressors (7, 8). The mammalian correlate of the “stressosome” is the HPA axis, as it provides a co-ordinated response to acute stress (9). The fundamental components of the central HPA axis are well known and include the corticotropin-releasing hormone (CRH)-secreting neurons of the paraventricular nucleus of the hypothalamus (PVN) (10) that stimulate pituitary adrenocorticotropic hormone (ACTH) and adrenal corticosterone (CORT) secretion (11).

Glucocorticoids are steroid hormones that are secreted by the adrenal glands and are important regulators of homeostasis in basal and stressful conditions. GCs exert their influence through two types of intracellular receptors the type I mineralocorticoid receptor and type II glucocorticoid receptor. Both receptors are expressed throughout the body and exert system-wide effects. In the brain, the high affinity type I mineralocorticoid receptor (also called aldosterone receptor in the kidneys), is expressed predominantly in the hippocampal formation and moderate expression is found in prefrontal cortex (PFC) and amygdala (12–14). The low affinity type II GRs are expressed throughout the brain with highest expression in the PVN and hippocampus and because of its lower affinity to cortisol it plays a key role in stress-related homeostasis when circulating levels of cortisol are high (14–17). GRs and MRs receptors reside in the cytoplasm and mediate classical genomic actions of GCs by acting as nuclear transcriptional activators and repressors (14, 18) and membrane bound GRs mediate the rapid actions of GCs (19, 20). GCs are thus ideally positioned to modulate responses to stress and be activated in the brain during healthy conditions, following acute stress and during adaptation of responses to chronic stress (4, 5, 21).

**Figure 1:**
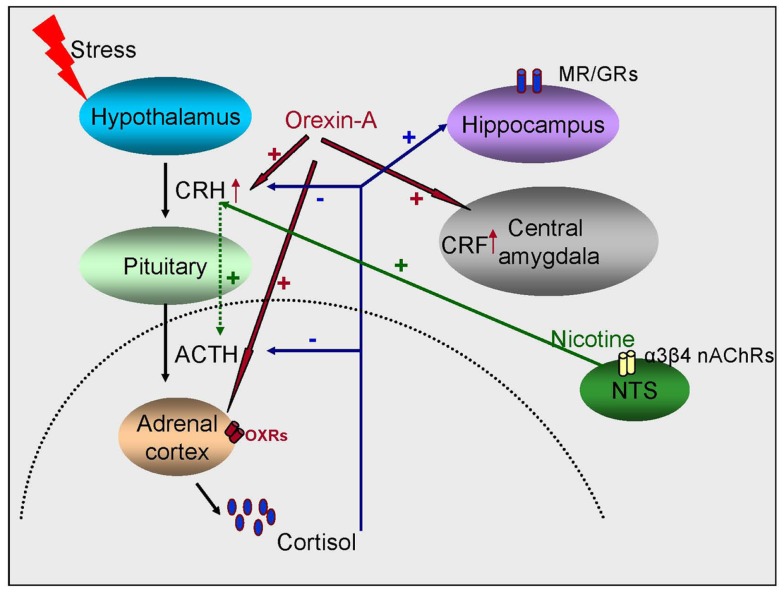
**Schematic representation of the interaction between glucocorticoids, orexins, and the cholinergic system in regulating stress responses**. Stress activates the release of glucocorticoids from the adrenal gland, which then feedback into the brain and target both the HPA axis and extra-hypothalamic sites like the hippocampus and the amygdala. Orexins also activate the HPA axis and lead to the production of glucocorticoids and stimulate the release of CRF from the PVN of the hypothalamus and the central amygdala. The third player are the nicotinic receptors (nAChRs) which indirectly regulate ACTH release by acting on the PVN.

Glucocorticoids provide inhibitory feedback responses over fast (seconds to minutes) and longer (hours to days) timescales (4, 18, 22–24). The rapid effects involve immediate reduction in miniature EPSC frequency upon application of corticosterone or dexamethasone (synthetic GC) in the PVN (25), and reduced ACTH and corticosterone levels, an effect not observed when membrane impermeable dexamethasone was used, indicating fast feedback inhibition (26). Similar rapid effects of corticosterone on mEPSC in the hippocampus have been observed (27, 28). Thus both short time scale (perhaps non-genomic) and longer time scale (genomic) actions of GC together mediate the inhibitory feedback control. The molecular and neurobiological processes that underpin passive and active resilience are being investigated and candidates are regulators of the HPA axis, molecules involved in the architecture of the synapse and signaling molecules associated with neural plasticity [reviewed by (2)]. GCs represent the end product of the HPA axis and influence many functions of the central nervous system, such as arousal, cognition, mood, sleep, metabolism, and cardiovascular tone, immune, and inflammatory reaction (Figure 1).

Repeated traumatic events induce long-lasting behavioral changes that affect cognitive, emotional, and social behaviors that ultimately provide an organism protection or survival. The ability to handle stress may depend on an individual’s HPA axis responsiveness that may in turn predict the likelihood of developing neuropsychiatric disorders such as addiction. However, under chronic stress this feedback becomes dysregulated leading to the variety of maladaptive syndromes, such as anxiety and various forms of depressive disorders (1, 5, 29–33) and addiction, including alcohol dependence (34). It has been shown that dysregulation of the HPA axis by chronic and uncontrollable stress leads to abnormal GC secretion (35, 36). GRs mediate adaptation to stress and regulate termination of the stress response through negative feedback at the level of the HPA axis (30–32). GCs can dynamically regulate tissue sensitivity in a stochastic manner (5) and control the response to chronic stress. GCs regulate tissue and organ sensitivity by modulating GRs signaling, ligand availability, receptor isoform expression, intracellular circulation, and promoter association (30–32).

## Glucocorticoid Receptors in Maladaptive Stress Responses: The Role of Changes in Plasticity in the Amygdala

The amygdala is a key brain region that is involved in processing stress, fear, and pavlovian conditioning, and is a site where neuroendocrine signals stimulated by fear and stress interact. It has been proposed that the balance between hippocampal and amygdalar learning is important for determining behavioral stress coping choices. Chronic restraint stress increases dendritic growth and spine density in the basolateral amygdala (BLA) and is in contrast to its role in the hippocampus. The changes in the hippocampus return to baseline during recovery, whereas those in the amygdala are long lasting (37). Neurotrophic factors like BDNF mediate the stress-induced alternations in these brains regions. A recent study demonstrated that increased levels of BDNF are found in response to chronic stress in the BLA, whereas decreased levels were observed in the hippocampus (38). Animals which escape from aggressive interactions seem to have a more robust BDNF expression profile in the hippocampus and less in the amygdala, while the opposite behavior (of stay and face the opponent) have the opposite effect (39). Thus stress activates neurotrophic factors in different brain regions and is thought to be mediated by the GR system. Mice with a targeted genetic deletion of the GR, specifically in the central nucleus of the amygdala (CeA) but not in the forebrain have decreased conditioned fear responses (40). In contrast, targeted forebrain disruption of GRs, excluding the CeA, did not. It is known that the GRs in BLA are involved in consolidation of emotionally arousing and stressful experiences in rodents and humans by interacting with noradrenaline. Human studies have demonstrated that interactions between noradrenergic activity and glucocorticoid stress hormones can bring out disruptions in the neural basis of goal-directed action to habitual stimulus-response learning (41). Recently, it was shown that following acute stress, LTP induction is facilitated in the BLA by both β-adrenergic and GRs activation (42). Taken together, there are circuit specific changes underlying learning during stressful conditions, animals that are susceptible to stress have greater increases in synaptic activity in fear-related circuits such as the amygdala compared to animals that are resilient to stress.

## Glucocorticoids Drive Changes in Plasticity in the Hippocampus and Cortical Regions in Response to Stress

Glucocorticoid receptors in the hippocampus control homeostasis during healthy conditions and then play a role in driving changes in plasticity in response to stressful conditions (43, 44). Early life experiences that ultimately control an individual’s HPA responsivity to stressful stimuli are modulated by GR gene expression in the hippocampus and frontal cortex (45). Hippocampal GRs play a role in the formation of long-term inhibitory avoidance memory in rats by inducing the CaMKIIα-BDNF-CREB-dependent neural plasticity pathways (46). In a separate study, chronic exposure to corticosterone resulted in impaired ability to learn response outcomes (47). Memory consolidation is thought to be mediated by the GR, while appraisal and responses to novel information is processed by the MR. Human and rodent studies suggest that under stressful conditions there is a switch from cognitive memory mediated by the hippocampus to habit memory mediated by the caudate nucleus (48, 49). In fact, mice deficient in MR receptors have impaired spatial memory, however they were rescued from further deterioration by stimulus-response memory following stress (50). Similarly, following an acute stressor, GRs are activated and induce synaptic plasticity in the PFC by increasing trafficking and function of NMDARs and AMPARs (51). Furthermore, when the MR was overexpressed in the forebrain of mice using a CAMkIIa promoter driven expression of HA-tagged human MR cDNA, the mice showed improved spatial memory, reduced anxiety without alteration in baseline HPA stress responses (52). There is mounting evidence that GCs participate in the formation of memories in specific circuits that govern stress responses and consequently responses to substances of abuse and alcohol.

## Glucocorticoids in the Development of Addiction

Chronic exposure to stress leads to alterations in the homeostatic functioning of GCs (29). Furthermore, there is significant dysregulation of the HPA axis following alcohol dependence. It has been shown that acute voluntary ethanol self-administration increases corticosterone levels, in contrast, long-term ethanol exposure in rodents results in a blunted response suggesting the alcohol dependence leads to dysregulation of the HPA axis (53). Transient overexpression of GR in young animals is both necessary and sufficient for bringing about profound changes in the transcriptome in specific brain regions leading to a lifelong increase in vulnerability to anxiety and drugs of abuse (54). The modified transcripts have been implicated in GR and axonal guidance signaling in dentate gyrus and dopamine receptor signaling in nucleus accumbens (NAc) (54). Furthermore, in some individuals, following exposure to stress and psychological trauma, GCs can promote escalated drug-taking behaviors and induce a compromised HPA axis. GCs can cross-sensitize with stimulant drug effects on dopamine transmission within the mesolimbic dopamine reward/reinforcement circuitry (55) and increase susceptibility to developing addictive behaviors (56–58) by increasing the synaptic strength of dopaminergic synapses (59). Importantly, the dopamine responses in the NAc core, but not the shell, have been shown to respond to fluctuating levels of GCs (60). Deficiencies in the GR gene in mice specifically in dopaminergic neurons expressing dopamine D1 receptors that receive dopaminergic input had decreased cocaine self-administration and dopamine cell firing (61). Acute exposure or binge-like ethanol exposure alter GC levels and promote PFC GC-regulated gene expression (62) and neurodegeneration that is dependent on type II GRs (63). GCs induce ethanol associated plasticity of glutamatergic synapses that have been proposed to underlie the development of ethanol dependence, reviewed in (64).

It has been shown that there is a correlation between acute alcohol withdrawal and downregulation of GR mRNA in the PFC, NAc, and bed nucleus of the stria terminalis (BNST), while protracted alcohol abstinence correlated with upregulated GR mRNA in the NAc core, ventral BNST, and CeA (65, 66), reviewed in (67). The transition from initial voluntary drug use to subsequent compulsive drug use has been proposed to reflect a switch from goal-directed to habitual control of action behavior (68). The investigators propose that acute stressors reinstate habitual responding to drug-related cues and repeated stress may promote the transition from voluntary to compulsory drug use. GCs are ideally positioned to regulate a diverse array of systems that modulate the development of addiction. In the following sections, we review the interplay between GCs and the orexinergic and cholinergic systems.

## The Orexinergic System

The most studied biological functions of orexins/hypocretins are in the central control of feeding, sleep, energy homeostasis, and reward-seeking. Orexin-A and orexin-B (also called hypocretin-1 and -2) interact with two orexin/hypocretin receptor subtypes, the Orexin_1_ Receptor (OX1R) and Orexin_2_ Receptor (OX2R) which bind to either or both orexin-A and orexin-B (69, 70). Initial discoveries on the role of orexins came about with identification of deficiencies in the genes either encoding orexin or the OX2R receptor resulting in canine narcolepsy, implicating the role of ORX/Hcrt system in the regulation of sleep and wakefulness (71, 72). Orexin-A and orexin-B have been shown to increase food intake that is blocked by selective antagonists (73, 74). In addition, orexinergic fibers innervate various brain regions involved in energy homeostasis, such as the ventromedial hypothalamic nucleus, the arcuate nucleus, and the PVN of the hypothalamus (75). Orexins regulate autonomic functions, such as regulation of blood pressure and heart rate (76). Thus these neuropeptides are in a unique position to respond to stress.

## Role of Orexins in Stress and Activation of the HPA Axis

**Figure 2:**
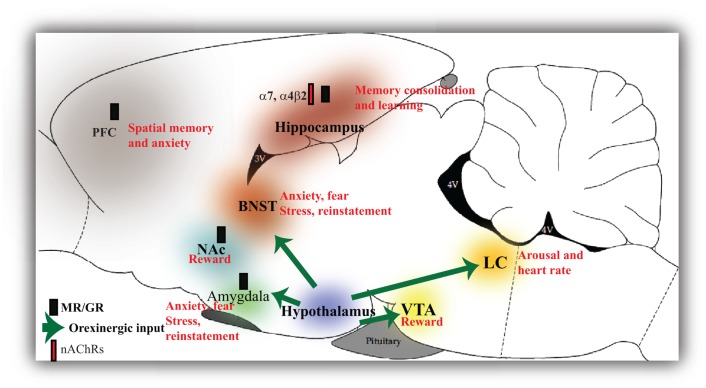
**Glucocorticoid, orexinergic, and cholinergic activation of the brain regions involved in stress and drug addiction**. Glucocorticoid receptors in the hippocampus and amygdala mediate the effects of stress and consolidation of fearful memories. GCs also modulate alcohol withdrawal in the prefrontal cortex (PFC), nucleus accumbens (NAc), and bed nucleus of the stria terminalis (BNST). Glucocorticoids (GCs) in the hippocampus also negatively regulate the hypothalamus thereby providing a central feedback mechanism. Orexins produced in the hypothalamus activate reward pathways such as the ventral tegmental area (VTA) and the NAc and brain regions involved in stress, fear, and anxiety such as the amygdala and BNST and regulate cardiovascular tone through the locus coeruleus (LC). Both GCs and orexins play similar roles in brain regions implicated in stress and reward. Glucocorticoids have been shown to directly inhibit nicotinic receptor (nAChR) activity in the hippocampus that exert an inhibitory effect on the HPA axis. The nAChRs seem to differentially orchestrate responses to stress.

Arousal is an important element of the stress response and the orexin system is a key component of the response to stress. Projections from perifornical nucleus and the dorsomedial nucleus of the hypothalamus are also implicated in addictive behaviors, however their role in arousal and concomitant stress has been the main focus (77). Orexins modulate the HPA axis in response to different stressful stimuli. Prepro-orexin mRNA expression was increased in the lateral hypothalamus (LH) in young rats following immobilization stress and in adult rats following cold stress (78). OX-A activates the HPA axis inducing secretion of ACTH and corticosterone (79). OX-A, but not OX-B, increases glucocorticoid secretion from rat and human adrenal cortices by direct stimulation of adrenocortical cells via OX1R coupled to the adenylate cyclase-dependent cascade (79) (Figure 1). Intracerebroventricular (I.C.V) administration of OX-A enhanced ACTH and corticosterone release (80–82). It has been proposed that orexin neurons play an integrative role that links autonomic responses to arousal and/or vigilance during the fight-or-flight response (83) (Figure 2).

## Role of Orexins in Addiction

Along with the many functions performed by orexins, the most intriguing is their role in the reward system. Orexin containing neurons project from the LH to the ventral tegmental area (VTA) and NAc, the brain regions that comprise the mesolimbic “reward pathway” (84–86). OXRs have recently been implicated in the motivational drive for addictive substances such as morphine, cocaine (87–91), and alcohol (92–97). The OX1R plays a specific role in ethanol self-administration, cue, and stress-induced relapse, reviewed in (98) with a more limited role for OX2R being shown (99). The orexin system has also been implicated in relapse to drug use. The OX1R plays a role in foot-shock stress-induced reinstatement of cocaine (100, 101) and cue and yohimbine induced reinstatement of ethanol-seeking (94, 96, 102).

The central amygdaloid projections regulate the HPA axis and innervate orexin containing neurons in the lateral hypothalamus. The extended amygdala which includes the CeA, BNST, and the NAc are critical brain areas that process emotional behaviors such as anxiety, fear, stress, and drug addiction. In particular, the CeA and BNST have been shown to play an important role in anxiety-related behaviors and voluntary ethanol consumption (103). The extended amygdala, including the CeA, has been shown to play a critical role in the reinstatement behavior to drugs of abuse. Inactivation of the CeA, but not the BLA, prevents foot-shock-induced reinstatement of cocaine-seeking (104). Dense orexinergic innervation is also observed in all these brain regions (76, 105, 106). These brain regions also express stress peptides such as corticotrophin releasing factor (CRF) and anti-stress peptides such as neuropeptide Y (NPY). Both these neuropeptides have opposing actions in the CeA and regulate ethanol consumption. OX-A infusions into the BNST produce anxiety like responses as measured by social interaction test and elevated plus maze test and the effect is mediated by NMDA receptors (107). A recent study also demonstrated that yohimbine activates orexinergic responses, but not adrenergic receptor activity, and depressed excitatory neurotransmission in the BNST that contributed to reinstatement of extinguished cocaine CPP (108). Thus the orexinergic system is involved in mediating stress-induced drug-seeking behavior as it recruits multiple brain regions involved in processing stressful stimuli and addictive behaviors. It is essential to understand the contribution of orexins in the overlap between stress and reward systems. Identifying circuits that mediate stress-induced relapse to drug abuse will be necessary in order to develop targeted pharmacotherapeutic approaches for stress-induced drug relapse. The dual orexin receptor antagonist, suvorexant (109) has successfully completed phase III clinical trials in treating primary insomnia and is currently under FDA review. If approved, this will be the first FDA orexin antagonist available for treating sleep-disorders and has the potential to be repurposed for its efficacy in treating stress and addictive disorders.

## Interactions between the Cholinergic System and HPA Axis

Allostasis, a process by which homeostasis is regained after stress, occurs by the interaction between the PFC, amygdala, and the hippocampus via the HPA axis (110–113). In this process a number of neurotransmitters and neuromodulators such as acetylcholine, glutamate, and GABA, have been shown to be differentially modulated. Here, we review the involvement of the components of the cholinergic pathway in reacting to, sustaining, and even exacerbating stress.

Components of the cholinergic pathway are – the ligand, acetylcholine (ACh); the enzyme responsible for the breakdown of acetylcholine, acetylcholinesterase (AChE); the enzyme involved in synthesizing ACh, choline acetyltransferase (ChAT); and, the acetylcholine receptors, nicotinic acetylcholine receptor (nAChR), and muscarinic acetylcholine receptor (mAChR). We are focusing specifically on the nicotinic receptor – nAChR – in relation to the cholinergic response to stress. By focusing on the *nAChR-cholinergic pathway*, it is not our purpose to suggest that nAChR is the only or a more important player mediating responses to stress. Rather, it is intended that this review highlights the interactions of the glucocorticoid pathway (mediated via the HPA) and the nAChR-cholinergic pathway in relation to stress.

It is well known that the nAChRs are involved in learning and memory (114, 115). Additionally, the negative effects of chronic stress on memory are also well established (116, 117). Indeed, as early as 1968, the hippocampus was recognized as a target structure for stress hormones (118) with observations that acetylcholine release into the hippocampus (119, 120) increased under various stress models (121). Transgenic mouse knock-out models have shown the importance of the α4 (122), β3 (123), and β4 (124) nAChR subunits in mediating the anxiogenic effects of stress. Furthermore, the α5 and β4 knock-out mice are less sensitive to nicotine (125, 126), a potent anxiolytic agent (127–129) at lower doses (130). Indeed, the α7 and α4β2 nAChRs, which are the primary targets of nicotine, have been shown to provide a nicotine-mediated neuroprotective effect in stress-induced impairment of hippocampus-dependent memory (131). The hippocampus has been shown to exert an inhibitory effect on the HPA axis (132–136), thus lowering stress. Taken together, the nAChR seem to differentially orchestrate responses to stress via its various subunits.

Activation of the stress response is due to the cascading efflux of CRH, ACTH, and cortisol. Nicotine, a potent ligand at nAChRs, in relatively high doses (2.5–5.0 μg/kg) has been shown to produce a dose-dependent increase in ACTH (137), and its antagonist, mecamylamine, has been shown to block nicotine-stimulated ACTH release (137, 138). In the brain, the region responsible for the CRH-mediated ACTH release is the parvocellular region of the PVN (pcPVN) of the hypothalamus (139, 140). It has, however, been shown that nicotine mediates ACTH release indirectly, via the nicotinic receptors on the nucleus tractus solitarius (NTS) (141, 142). The NTS subsequently mediates action potentials via various afferents to the pcPVN (143, 144). The nAChR in the NTS are found pre-synaptically on glutamatergic projections to the pcPVN (145, 146). Further, the nAChR subunits implicated in the nicotine-mediated effects of ACTH in this pathway are the β_4_-containing nAChRs (most likely α_3_β_4_^∗^) but not the α_4_β_2_ as determined by measurements of mEPSCs in the presence of DHβE, a potent α_4_β_2_ inhibitor or cytisine, a potent β_4_^∗^-nAChR agonist (146). Therefore, while the α_4_β_2_ and α_7_ nAChR subunits modulate nicotine-mediated roles elsewhere (131), in the NTS it is a different subtype (146), pointing yet again to a nAChR-based differential modulation to stress (Figure 1).

## Glucocorticoid Interactions with the Cholinergic System

Glucocorticoids have been shown to directly inhibit nAChR activity (147–149). This is supported by the fact that stress causes a down regulation of the nAChR in the rat cerebral cortex and midbrain (150). Additionally, steroid antagonists have been shown to upregulate nAChR expression (151). That GCs can directly affect nAChR activity via receptor binding or alteration of expression levels can be explained by the presence of glucocorticoid response elements (GRE) on genes transcribing the α_7_ subunit of the nAChR – CHRNA7 (152). Indeed, GREs have also been identified on genes for ChAT (153) and AChE (154), components of the cholinergic pathway. Further research is required to study the precise effects of these GREs in this pathway along with investigating if these GRE are also present on other nAChR genes.

Other components of the cholinergic pathway too have been shown to be affected by stress. AChE, responsible for the timely degradation of ACh, has been shown to be regulated via alternative splicing thus modifying neurotransmission (155). Indeed, miRNA post-transcriptional modification of AChE from its usual AChE-S to the read-through form AChE-R alters cholinergic transmission (156). Additionally, post-transcriptional modulation of AChE, again via miRNA, causes hippocampal-related cognitive defects (157). As stated earlier, AChE expression is controlled at the genomic level via the GRE (154) as is ChAT (153). Also, ChAT protein levels were shown to decrease due to chronic stress (158). At the epigenetic level, there is stress-induced epigenetic transcriptional memory of AChE via HDAC4 (159). Interestingly, in this study a GRE was also identified on HDAC4 (159), suggesting a direct epigenetic effect of stress on AChE. All these results point to a multi-faceted mechanism whereby the stress-induced cholinergic response is regulated without the over-articulation of its response that would undoubtedly lead to various stress-related neuropathologies such as PTSD (160, 161), alcohol addiction (162, 163), and addiction to other substances of abuse (164, 165).

In summary, the involvement of the different subtypes of the nAChR in different regions of the brain along with modulation of the cholinergic pathway at various stages such as transcriptional, post-transcriptional, and epigenetic modifications, point to a finely modulated system both temporally and spatially that is attuned to respond to the various stressors that we are faced with in our daily lives. Lastly, while this review has focused on the nAChR and the cholinergic pathway, the involvement of the muscarinic receptor and a myriad other neural circuits cannot be understated. Indeed the ultimate goal of this field of research is to understand sufficiently the intricate interplay between the various pathways and neural circuits that ultimately will enable the alleviation of stress-induced morbidity via development of more effective pharmacotherapeutic strategies against stress.

## Pharmacotherapeutic Strategies

Ample evidence exists to demonstrate that type II GRs are important therapeutic targets for the treatment of disorders that result from maladaptive stress responses. Mifepristone, also known as RU486, is a derivative of the 19-norprogestin norethindrone and potently competes with type II GRs and progesterone receptors (PRs). Mifepristone has been shown to reduce reinstatement of ethanol-seeking and escalated drinking in two different animal models (66, 166). Furthermore, mifepristone has been shown to be effective at reducing the self-administration of amphetamine (167), cocaine (168, 169), morphine (170), and ethanol (57, 66, 162, 166, 171–175). A recent study also demonstrates the effectiveness of mifepristone in reducing withdrawal symptoms of alcohol (176). The anti-glucocorticoid activity of mifepristone has made it a potential treatment for Cushing’s syndrome (177) and neurological and psychological disorders (178–183). Mifepristone offers a promising way to temporarily reset the stress response system that has become maladapted following chronic and long-term alcohol consumption.

## Conclusion

Learning to cope with life and/or stress or learning to be susceptible to stress involves dynamic regulation of plasticity in brain circuits that govern stress response pathways. As the brain can be remodeled by experience and neural circuits are adaptable and dynamically regulated, this suggests it is possible to change the brain or learn how to cope with stress and overcome addiction and learn to become more resilient. The molecular pathways and circuits that govern resilience are gradually being uncovered and this will provide opportunities for identifying novel strategies that overcome the impact of addiction on the brain combined with possible novel pharmacotherapeutic strategies that target pro-resilience pathways. In this review, we focused on the role of glucocorticoid hormones, as they have the capacity to provide system-wide feedback during acute and chronic stress and provide a way forward to interrogate and reset brain networks. Understanding the molecular mechanisms that govern mechanisms that the brain utilizes to protect from the deleterious effects of stress will provide exciting new avenues in neuroscience.

## Conflict of Interest Statement

The authors declare that the research was conducted in the absence of any commercial or financial relationships that could be construed as a potential conflict of interest.
